# Signaling Transduction Pathways and G-Protein-Coupled Receptors in Different Stages of the Embryonic Diapause Termination Process in *Artemia*

**DOI:** 10.3390/cimb46040229

**Published:** 2024-04-20

**Authors:** Tong Hao, Zhentao Song, Mingzhi Zhang, Lingrui Zhang

**Affiliations:** Tianjin Key Laboratory of Animal and Plant Resistance, College of Life Sciences, Tianjin Normal University, Tianjin 300387, China; a953835912@gmail.com (Z.S.); 18404964001@163.com (M.Z.); ztt11235813@163.com (L.Z.)

**Keywords:** embryonic diapause termination, signal transduction, GPCR, *Artemia* cyst, high-throughput sequencing

## Abstract

*Artemia* is a widely distributed small aquatic crustacean, renowned for its ability to enter a state of embryonic diapause. The embryonic diapause termination (EDT) is closely linked to environmental cues, but the precise underlying mechanisms remain elusive. In this study, ATAC-seq and RNA-seq sequencing techniques were employed to explore the gene expression profiles in *Artemia* cysts 30 min after EDT. These profiles were compared with those during diapause and 5 h after EDT. The regulatory mechanisms governing the EDT process were analyzed through Gene Ontology (GO) enrichment analysis of differentially expressed genes. Furthermore, the active G-protein-coupled receptors (GPCRs) were identified through structural analysis. The results unveiled that the signaling transduction during EDT primarily hinges on GPCRs and the cell surface receptor signaling pathway, but distinct genes are involved across different stages. Hormone-mediated signaling pathways and the tachykinin receptor signaling pathway exhibited heightened activity in the ‘0–30 min’ group, whereas the Wnt signaling pathway manifested its function solely in the ‘30 min–5 h’ group. These results imply a complete divergence in the mechanisms of signal regulation during these two stages. Moreover, through structural analysis, five GPCRs operating at different stages of EDT were identified. These findings provide valuable insights into the signal regulation mechanisms governing *Artemia* diapause.

## 1. Introduction

*Artemia*, more commonly recognized as brine shrimp, is a small aquatic crustacean inhabiting diverse saline environments across the world. Among its notable adaptations, *Artemia* show a remarkable capability to undergo embryonic diapause—a phenomenon observed in numerous plants, insects, and mammals [1,2]. Diapause serves as a biological strategy enabling embryos to enter a state of suspended animation or dormancy until environmental conditions become conducive to hatching and subsequent survival [3]. This mechanism assumes a crucial role in the endurance and perpetuation of *Artemia* populations within unpredictable and frequently hostile aquatic habitats.

The onset of diapause is primarily triggered by adverse environmental conditions, such as fluctuations in temperature, salinity, and oxygen levels, or the presence of predators [4]. Upon detecting these unfavorable conditions, embryos of *Artemia* defer their developments to avoid hatching into potentially inhospitable surroundings. Throughout diapause, *Artemia* embryos undergo various physiological transformations, notably a reduction in metabolic activity. Entering a state of metabolic arrest, they conserve energy to endure adverse conditions for prolonged periods [5]. A high-energy substance called diguanosine (Gp4G) is preferentially utilized over conventional energy storage substances such as trehalose, glycogen, and glycerol during diapause [6]. The duration of diapause varies depending on environmental conditions. In some instances, diapause may persist for months or even years until the external environment become more conducive. After diapause termination, metabolic processes within the embryo are reactivated, leading to resumption of protein synthesis, cell division, and overall growth.

The mechanisms governing EDT in *Artemia* are complex and involve a combination of factors including environmental cues, hormonal fluctuations, and the engagement of specific genes and molecular pathways. The primary trigger for diapause termination is the recognition of improved environmental conditions, such as stable temperatures, optimal salinity levels, and the absence of stressors or predators [7,8]. Upon detection of these favorable cues, the embryo receives signals to resume development. Hormonal regulation likely constitutes another critical aspect of diapause termination. A diapause hormone receptor-like gene (Ar-DHR) has been identified in *Artemia*. It is located on the cell membrane of the pre-diapause cyst but is then found in the cytoplasm of the diapause cyst [9]. Changes in hormone levels within the embryo likely play a role in initiating the exit from diapause and promoting subsequent development. Additionally, genetic and molecular factors contribute to the termination of diapause in *Artemia*. Specific genes are either activated or suppressed to coordinate the resumption of embryonic development. Jia et al. [10] discovered a correlation between EDT and *Artemia* DEK (Ar-DEK), a nuclear factor protein. This correlation was observed through an increase in euchromatin and a decrease in heterochromatin. Furthermore, their research identified a connection between the Wnt signaling pathway and the EDT process in *Artemia*. Lin’s research team identified several genes crucial for diapause termination and cell cycle regulation during early embryonic development in *Artemia*. These genes include apoptosis inhibitor 5 (API5) [11], P53 and DNA damage-regulated gene 1 (pdrg1) [12], retinoblastoma binding protein 4 (RBBP4) [13], and glycerol kinase (GK) [14]. The activation of embryos is a swift process, with alterations in transcription levels observable within 30 min after dormancy is disrupted, peaking after 5 h [15,16].

The mechanism of *Artemia* EDT is a finely tuned survival strategy, enabling these small crustaceans to optimize the likelihood of their offspring hatching and flourishing in the dynamic aquatic environments they inhabit. Comprehensive investigation into the diapause termination mechanism aids researchers in comprehending how *Artemia*’s physiological activities are regulated in response to environmental shifts. However, to date, only a handful of environmental factors and genes have been identified as influencing the EDT process. The molecular mechanism of EDT remains elusive, particularly for the signal transduction process.

To gain a deeper insight into the signal transduction process of EDT, we conducted an analysis of the gene expression profile of *Artemia* cysts at 30 min after EDT, which represents the initial phase of the EDT process, using ATAC-seq and RNA-seq techniques. The profile was compared with that of the diapause stage and 5 h after EDT to validate the differentially expressed genes (DEGs) related to the signal transduction pathways and GPCRs in different stages of EDT through GO enrichment analysis and structural examination. The identification of signal transduction pathways and GPCRs provides crucial insights for further exploring the EDT mechanism in *Artemia*.

## 2. Materials and Methods

### 2.1. Artemia Hatching and Culture

*Artemia parthenogenetica* cysts (provided by the Asian Regional *Artemia* Reference Center, ARARC, Tianjin, China) were collected at Ebinur Lake and reactivated after dehydration and refrigeration treatment to break diapause. At 30 min after diapause breaking, the dry cysts were thoroughly rehydrated in ice-cold 30‰ artificial seawater and reactivated in 30‰ artificial seawater at 28 °C under continuous illumination. The reactivated cysts were collected, placed in liquid nitrogen immediately, and then preserved in a −80 °C refrigerator. The cell activity of the samples was assessed with a Trypan blue assay and quantified.

### 2.2. Experiment Design for ATAC-Seq and RNA-Seq

**Figure 1:**
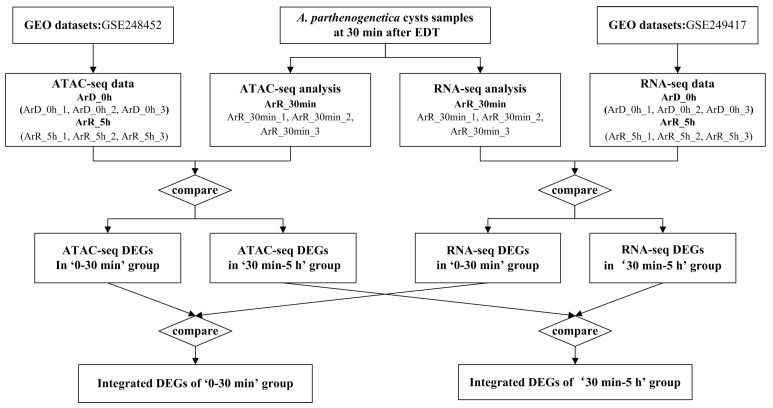
Illustration of the experimental design.

The *A. parthenogenetica* cysts collected 30 min after EDT were categorized as the ArR_30min group. A total of three biological replicates were collected, labeled as ArR_30min_1, ArR_30min_2, and ArR_30min_3. In preparation for subsequent sequencing, each of the samples was divided into two parts: one for ATAC-seq and the other for RNA-seq analysis. The sequencing results of ArR_30min were then compared with the ATAC-seq and RNA-seq data of *A. parthenogenetica* cysts collected at the diapause stage (ArD_0h group) and 5 h after EDT (ArR_5h group), all obtained from the same batch of cyst samples. These data were sourced from the GEO database under the accession numbers GSE248452 (ATAC-seq data for ArD_0h and ArR_5h groups) and GSE249417 (RNA-seq data for ArD_0h and ArR_5h groups). Each group comprised three biological replicates, labeled as ArD_0h_1, ArD_0h_2, ArD_0h_3, ArR_5h_1, ArR_5h_2, and ArR_5h_3. The comparison of ATAC-seq datasets resulted in the ATAC-seq DEGs for ArR_30min vs. ArD_0h (referred to as the ‘0–30 min’ group) and ArR_5h vs. ArR_30min (referred to as the ‘30 min–5 h’ group). Similarly, the comparison of RNA-seq datasets resulted in the RNA-seq DEGs for the ‘0–30 min’ group and ‘30 min–5 h’ group. To identify the genes with consistent expression patterns and chromatin accessibility, the DEGs in ATAC-seq and RNA-seq of the ‘30 min–5 h’ group were compared to identify the integrated DEGs (IDEGs) of the ‘0–30 min’ group. Similarly, the DEGs in ATAC-seq and RNA-seq of the ‘30 min–5 h’ group were compared to identify the IDEGs of the ‘0–30 min’ group. An illustration of the experimental design is shown in Figure 1.

### 2.3. ATAC Sequencing and Analysis

ATAC-seq was conducted as previously reported [17,18,19]. In brief, nuclei were extracted from each sample, and the nuclei pellet was re-suspended in Tn5 transposase reaction mix. The transposition reaction was then incubated at 37 °C for 30 min. Following transposition, equimolar amounts of adapter 1 and adapter 2 were added. Subsequently, PCR was performed to amplify the libraries. After PCR amplification, the libraries were purified using AMPure beads, and their quality was assessed with a Qubit instrument. The index-coded samples were clustered on a cBot Cluster Generation System using the TruSeq PE Cluster Kit v3-cBot-HS (Illumina, San Diego, CA, USA) according to the manufacturer’s instructions. Once cluster generation was completed, the library preparations were sequenced on an Illumina HiSeq platform (Illumina, San Diego, CA, USA), producing 150 bp paired-end reads.

After sequencing, Nextera adaptor sequences were trimmed from the reads using skewer (0.2.2). Subsequently, these reads were aligned to the *A. franciscana* genome (NCBI assembly ID ASM3288406v1) using BWA (version 0.7.12-r1039, Cambridge, UK) [20] with standard parameters. Following alignment, the reads were filtered for high quality based on criteria including a MAPQ ≥ 13 (i.e., *p* ≤ 0.05), exclusion of mitochondrial chromosomes, and retention of properly paired reads longer than 18 nucleotides. Data quality was assessed using FastQC (http://www.bioinformatics.babraham.ac.uk/projects/fastqc/, accessed on 10 May 2023, Cambridge, UK), and peak calling was performed using Macs2 software (version 2.2.7.1, Boston, MA, USA) [21]. Simulations of peaks called per input read utilized aligned and de-duplicated BAM files without any additional filtering.

The enrichments of peaks in the ArR_30min group were compared with those of the ArD_0h and ArR_5h groups, respectively. Differential peak analysis was carried out using the DESeq2 R package (version 1.20.0, Boston, MA, USA) [22], where peaks with |log2.FoldEnrich| > 1 were identified as differential peaks. The ChIPseeker R package (version 1.38.0, Hong Kong, China) [23] was employed to analyze the distribution of peaks in different functional regions, following a designated priority order: promoter, UTR, exon, intron, downstream TTS, and distal intergenic regions. In cases where a peak spanned both the promoter region of one gene and the UTR region of another gene, the priority order determined it as belonging to the promoter region rather than the UTR region. For Gene Ontology (GO) and Kyoto Encyclopedia of Genes and Genomes (KEGG) annotation of genes associated with differentially expressed peaks, GOseq (version 4.10.2, Parkville, Australia) [24] and KOBAS (version 3.0, Beijing, China) [25] software were utilized, respectively.

### 2.4. RNA Sequencing and Analysis

The mRNA-Seq experiments were conducted by Novogene (Beijing, China) using Illumina high-throughput sequencing technology. Initially, total RNA was isolated from *Artemia* cysts using TRIzol reagent (Thermo Fisher Scientific, Waltham, MA, USA), followed by treatment with RNase-free DNase I to eliminate any potential genomic DNA contamination. RNA integrity was assessed using the RNA Nano 6000 Assay Kit on the Bioanalyzer 2100 system (Agilent Technologies, Santa Clara, CA, USA). Sequencing libraries were generated with the NEBNext^®^ Ultra™ RNA Library Prep Kit for Illumina^®^ (NEB, Beverly, MA, USA). Library quality was evaluated using the Agilent Bioanalyzer 2100 system. Index-coded samples were clustered on a cBot Cluster Generation System with a TruSeq PE Cluster Kit v3-cBot-HS (Illumina). Subsequently, the prepared libraries were sequenced on an Illumina NovaSeq platform, yielding 150 bp paired-end reads.

After sequencing, the original image data were processed into sequencing data via base calling to generate raw reads. Clean data, including clean reads, were obtained by filtering out raw reads containing adapters or poly-N sequences or exhibiting low quality. Subsequently, the quality of clean data was assessed in terms of Q20, Q30, and GC content. All subsequent analyses were conducted using high-quality clean data. The *A. franciscana* genome (NCBI assembly ID ASM3288406v1) served as the reference genome for genome mapping. An index of the reference genome was constructed using Hisat2 (version 2.0.5, Baltimore, MD, USA) [26], and paired-end clean reads were aligned to the reference genome using the same software. Reads mapped to each gene were quantified using FeatureCounts (version 1.5.0-p3, Parkville, Australia) [27]. The FPKM (fragments per kilobase of transcript sequence per million base pairs sequenced) value for each gene was calculated based on its length and the number of reads mapped to it.

Gene expression levels in the samples from the ArR_30min group were compared with those from the ArD_0h and ArR_5h groups. To identify DEGs, a differential expression analysis was conducted using the DESeq2 R package (1.20.0). The resulting *p*-values were adjusted using the Benjamini and Hochberg method to control the false discovery rate. Genes with an adjusted *p*-value (Padj) ≤ 0.05 and |log2.Fold_change| > 1 were considered differentially expressed. Enrichment analysis of GO and KEGG for the DEGs was performed using GOseq (version 4.10.2, Parkville, Australia) [24] and KOBAS software (version 3.0, Beijing, China) [25], respectively. The ATAC-seq and RNA-seq data were deposited in the GEO database with the accession numbers GSE254934 and GSE254935.

### 2.5. Integration Analysis of ATAC-Seq and RNA-Seq

The expression profiles of the DEGs in the ATAC-seq and RNA-seq results were compared. Specifically, the up-regulated DEGs in RNA-seq were compared with the genes associated with up-regulated peaks in ATAC-seq, while the down-regulated DEGs in RNA-seq were compared with the genes associated with down-regulated peaks in ATAC-seq. In cases where a gene was associated with both up-regulated and down-regulated peaks in the ATAC-seq results, its expression profile consistent with the RNA-seq data was retained. Genes exhibiting consistent expression profiles in both methods were subjected to GO and KEGG enrichment analysis.

### 2.6. Structural Analysis of Candidate GPCR Proteins

To further identify candidate GPCR genes, the transmembrane helix (TMH) structure, secondary structure, and tertiary structure of the proteins encoded by these candidate genes were analyzed. The TMH structure of the proteins was predicted using TMHMM (https://services.healthtech.dtu.dk/service.php?TMHMM-2.0, accessed on 2 February 2024) [28]. The secondary and tertiary structures of the proteins were predicted using PredictProtein (https://predictprotein.org/, accessed on 2 February 2024) [29] and SWISS-MODEL (https://swissmodel.expasy.org/, accessed on 2 February 2024) [30], respectively.

## 3. Results

### 3.1. Landscape of Accessible Chromatin Regions in Artemia Cysts Based on ATAC-Seq

**Figure 2:**
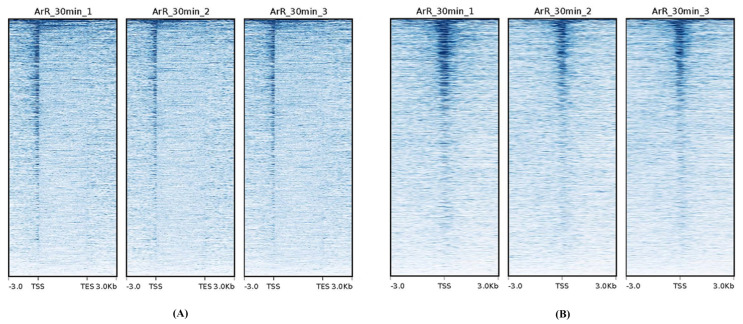
Heatmap of mapped reads’ distributions across the gene bodies (**A**) and TSSs (**B**).

ATAC-seq was performed to examine the landscape of genomic chromatin accessibility in the samples of the ArR_30min group. Raw data from ATAC-seq underwent initial trimming to obtain clean data for subsequent analysis. Details of data trimming and the quality control for both the raw and clean data are provided in Table 1. Clean data were then aligned to the reference genome. In the ATAC-seq analysis, the number of reads that uniquely and non-duplicatedly mapped to the reference genome was utilized. Accordingly, the clean data were filtered to select uniquely mapped reads, and any duplicated reads mapping to the same reference sequence were eliminated. The results of genome mapping are summarized in Table 2. The mappabilities of all samples exceeded 93%, indicating a high mapping ratio. On average, 53.4% of the uniquely mapped reads remained after deduplication across all samples. The distribution of mapped reads across gene bodies and transcriptional start sites (TSSs) revealed a high signal intensity in the TSS region, indicative of the high quality of the ATAC-seq data (Figure 2).

**Figure 3:**
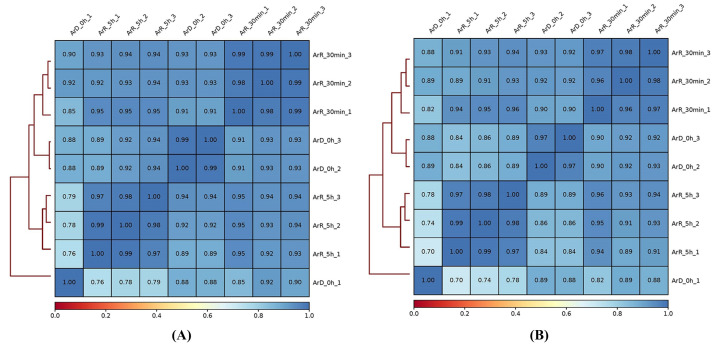
Pearson (**A**) and Spearman (**B**) correlations of reads in the samples.

To further assess the quality of the ATAC-seq data, Pearson and Spearman correlation analyses were conducted among samples of the ArD_0h, ArR_30min, and ArR_5h groups based on the signals of merged peaks from all samples. The results are displayed in Figure 3. A correlation coefficient closer to 1 indicates a higher degree of similarity in expression patterns between samples. As depicted in Figure 3, the samples in the ArR_30min group exhibited a high degree of similarity, with coefficients exceeding 0.96, indicating strong correlations within this group. Moreover, the similarities between the three samples of the ArR_30min group and those of the ArD_0h and ArR_5h groups were so great that they were essentially identical, further confirming the high quality of the samples. Subsequent to read mapping, peak calling was performed, and the results of this analysis are presented in Table 2.

### 3.2. DEG Analysis of ATAC-Seq

**Figure 4:**
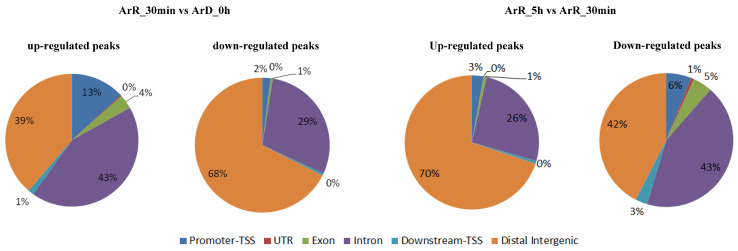
The genome-wide distribution of differential peaks.

The “FoldEnrich” values of each peak were compared among the ArD_0h, ArR_30min, and ArR_5h groups. A total of 6492 differential peaks were identified in the ‘0–30 min’ group, comprising 2584 up-regulated and 3908 down-regulated peaks. In the ‘30 min–5 h’ group, 6245 differential peaks were identified, with 4372 up-regulated and 1874 down-regulated peaks. The functional regions of each peak on the genome were annotated and categorized into promoter-TSS, UTR, exon, intron, downstream gene start site (TSS), and distal intergenic regions. The promoter-TSS region is typically enriched in the TSS. The distribution of functional regions among differential peaks is illustrated in Figure 4. Distal intergenic and intron regions accounted for the largest proportion of differential peaks. In the ‘0–30 min’ group, 13% of up-regulated peaks and 2% of down-regulated peaks were enriched in the promoter-TSS region. In contrast, in the ‘30 min–5 h’ group, only 3% of up-regulated peaks and 6% of down-regulated peaks were enriched in the promoter-TSS region.

**Figure 5:**
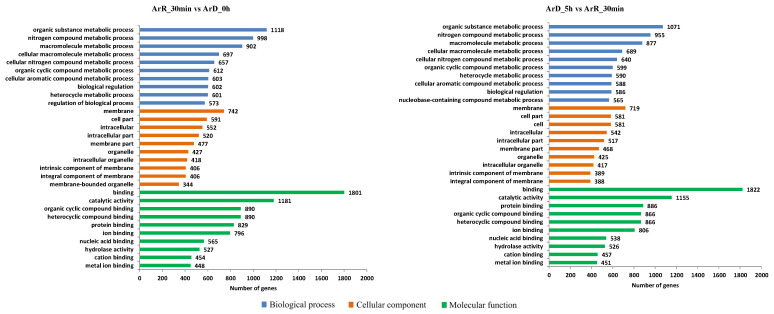
GO enrichment analysis of DEGs from ATAC-seq.

The 6492 differential peaks identified in the ‘0–30 min’ group were associated with 4261 genes, which were considered DEGs. Among these DEGs, 1880 were linked to up-regulated peaks, and 2381 were linked to down-regulated peaks. In the ‘30 min–5 h’ group, the 6245 differential peaks were related to 4312 genes, which were also considered DEGs. Among these DEGs, 2784 were associated with up-regulated peaks, and 1528 were associated with down-regulated peaks. GO enrichment analysis of the DEGs in the two groups revealed distinct patterns. In the biological process category, a significant number of DEGs were associated with organic substance, nitrogen compound, and macromolecule metabolic processes. Moreover, regulatory processes exhibited high activity in both groups. In terms of the cellular compartment and molecular function analysis, the DEGs were predominantly enriched in the membrane and cell parts, with binding and catalytic activity (Figure 5). The GO enrichment analysis of DEGs suggested that metabolic and regulatory processes in *Artemia* cells undergo significant changes within 5 h after EDT. Therefore, it is imperative to conduct a detailed analysis of the EDT process at different stages.

### 3.3. Gene Expression Profiles in Artemia Cysts Based on RNA-Seq

**Figure 6:**
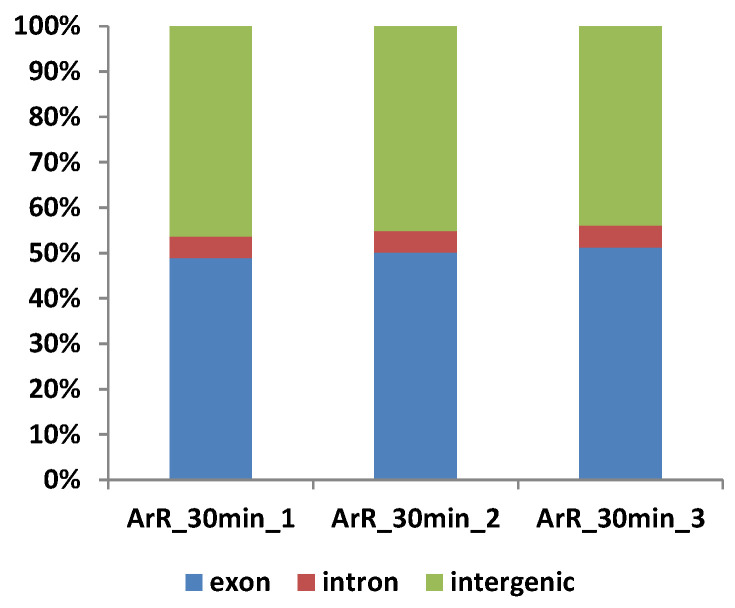
Distribution of mapped reads on *Artemia* genome.

Total RNA was extracted from the ArR_30min samples to obtain *Artemia* RNA-seq data. The original raw data underwent filtering, and the sequenced error rate and GC content distribution were assessed to obtain clean reads for subsequent analysis. After quality control, the clean reads were mapped to the reference genome. The results are presented in Table 3. The percentage of Q20 exceeded 97% for all samples, while that for Q30 exceeded 93%, indicating high sequencing quality. The GC percentages were consistent across all samples, ranging from 39.67 to 39.87%. The average percentage of mapped clean reads to the reference genome in ArR_30min samples was 89.20%, further demonstrating the robust quality of the sequencing results. Most of the mapped reads were distributed in the exonic and intergenic regions of the genome, with only a small portion (approximately 4.78%) located in intronic regions (Figure 6). Reads mapped to intronic regions may originate from precursor mRNA or introns retained due to alternative splicing events. Reads mapped to intergenic regions may be attributed to non-coding RNA (ncRNA), minimal DNA fragment contamination, or potential gaps in genome annotation.

Through the analysis of DEGs, a total of 4303 DEGs were identified in the ‘0–30 min’ group, with 2414 DEGs up-regulated and 1889 DEGs down-regulated. In the ‘30 min–5 h’ group, 5815 DEGs were identified, consisting of 2999 up-regulated and 2816 down-regulated DEGs.

**Figure 7:**
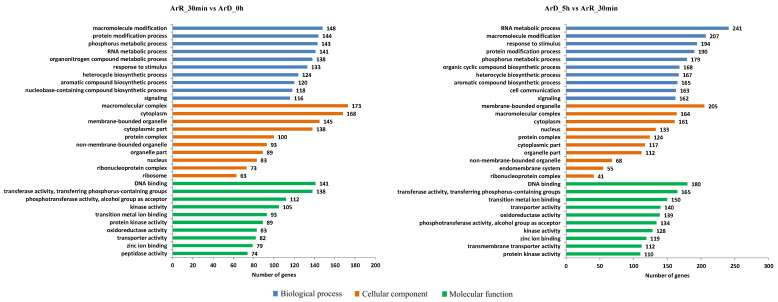
GO enrichment of DEGs from RNA-seq.

The GO enrichment analysis of these DEGs revealed similar patterns in both the ‘0–30 min’ and ‘30 min–5 h’ groups. In the biological processes category, these DEGs were primarily enriched in processes related to macromolecule modification, protein modification, various metabolic and biosynthetic processes, responses to stimuli, cell communication, and signaling processes. Cellular localization analysis indicated that these DEGs were predominantly located in membrane-bound organelles, macromolecular complexes, and the cytoplasm. Furthermore, these DEGs were often associated with DNA binding and transferase activity (Figure 7). The enriched annotations of the DEGs indicated substantial changes in metabolic processes following the reactivation of *Artemia* cysts, with increased cell communication potentially mediated by the signaling system.

### 3.4. Integration Analysis of ATAC-Seq and RNA-Seq

The genes associated with differential peaks from the ATAC-seq data were compared with the DEGs from the RNA-seq data. DEGs that were common and exhibited the same expression profile were referred to as IDEGs in the subsequent analysis. The IDEGs found in the ‘0–30 min’ and ‘30 min–5 h’ groups are presented in Table 4. In both groups, the number of up-regulated genes was higher than that of down-regulated genes, suggesting that within the initial 5 h of EDT occurrence, cellular activity continued to enhance. Cells stimulated metabolism and developmental processes by expressing a greater number of genes.

Although both groups had over 700 IDEGs each, they only shared 131 genes in common. Among these, 39 genes exhibited inconsistent regulation between the two groups, leaving only 92 genes that were common and consistently up-regulated or down-regulated in both groups. This indicates significant changes in the *Artemia* cyst cells during the first 30 min and 30 min to 5 h after the initiation of EDT, with substantial differences in metabolic and regulatory processes.

**Figure 8:**
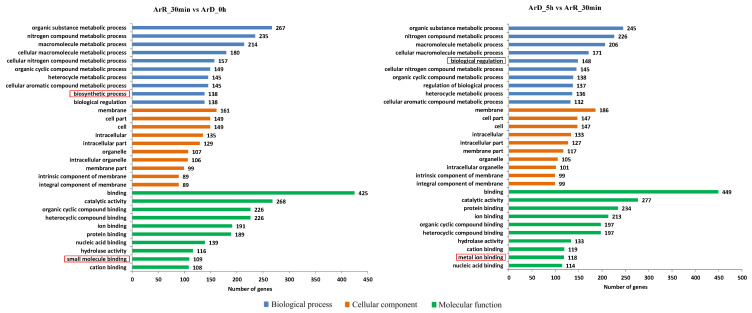
GO enrichment analysis of IDEGs. The main differences between the two groups were marked with red rectangles.

The results of the GO enrichment analysis for the IDEGs are shown in Figure 8. In terms of biological processes, these genes were primarily enriched in biological regulation and metabolic processes, including organic substance, nitrogen compound, and macromolecule metabolic processes. The distinction between the ‘0–30 min’ and ‘30 min–5 h’ groups lay in the fact that biosynthetic processes were more active in the former, while biological regulation was more prominent in the latter. This indicates that during the initiation of the EDT process, certain biological molecules related to reactivation and developments are synthesized initially, followed by a gradual increase in the activation of regulatory processes. Among the 92 DEGs that exhibited consistent regulation in both groups, 34 genes were enriched in metabolic processes, while only 17 genes were enriched in biological regulation processes. This suggests that changes in regulatory processes were more pronounced during the 30 min and 5 h periods of EDT compared to metabolic processes.

Regarding cellular components, the DEGs in both groups were predominantly located in the membrane and cell part. In terms of molecular function, the DEGs were primarily associated with binding and catalytic activity. The only difference observed was that the ‘0–30 min’ group had a higher number of genes related to small molecular binding, while the ‘30 min–5 h’ group had a higher number of genes related to metal ion binding (Supplementary File S1).

**Figure 9:**
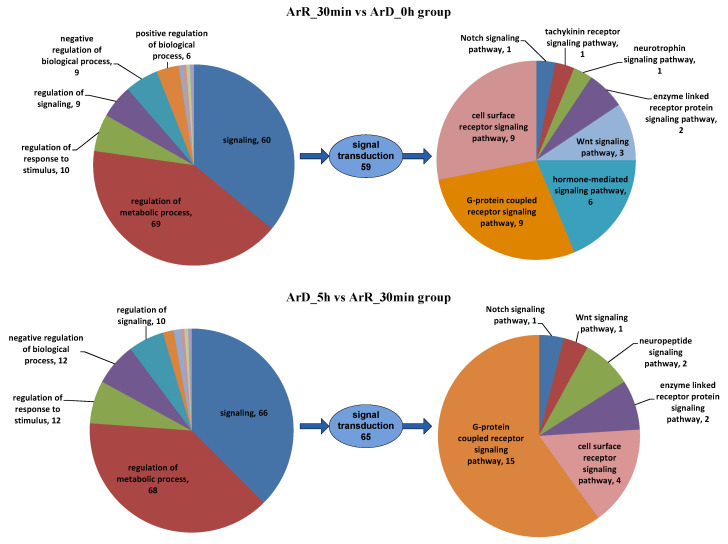
GO enrichment analysis of IDEGs in the regulation of biological process. Arabic numerals represent the number of genes in each GO item.

To further investigate the regulation of EDT, the distribution of DEGs involved in the “regulation of biological processes” (GO:0050789) in the two groups was analyzed. As depicted in Figure 9, in both groups, “regulation of cellular process” and “signaling” play predominant roles in the regulatory process. Within the “signaling” process, only eight genes were shared in both groups, indicating significant differences in the signaling regulation processes during the initial 30 min and 5 h of EDT. A commonality between these two stages is that the signaling genes in both groups are predominantly enriched in “signal transduction”, signifying the crucial role of signal transduction in the EDT and development of *Artemia* cysts.

Upon further analysis of the DEGs related to signal transduction, it was observed that they primarily function in the “G protein-coupled receptor signaling pathway” and the “cell surface receptor signaling pathway”. This suggests that *Artemia* primarily transmits signals through intracellular signaling pathways after receiving signals through cell surface receptors such as GPCRs. The difference lies in the fact that in the ‘0–30 min’ group, the hormone-mediated signaling pathway is enriched among the DEGs, indicating that hormones may play a crucial role at the outset of EDT. Among the DEGs involved in the G-protein-coupled receptor signaling pathway, 5 genes were identified to have GPCR activity (GO:0004930) in the ‘0–30 min’ group, and 10 genes exhibited GPCR activity in the ‘30 min–5 h’ group. These genes were thus considered as candidate GPCR genes that may play a significant role in the EDT and reactivation of *Artemia* development. However, it is noteworthy that there was no overlap in the genes with GPCR activity between the two groups, suggesting that the GPCR-associated signaling transduction processes within the first 30 min and 5 h after EDT may be entirely distinct.

### 3.5. Structural Analysis of Candidate GPCR Genes

The distinguishing feature of GPCRs is the presence of 7-TMHs. To further confirm the identity of candidate GPCR genes, the TMHs, secondary structures, and tertiary structures of the proteins encoded by the candidate GPCR genes were analyzed using TMHMM, PredictProtein, and SWISS-MODEL software, respectively. The results revealed that in the ‘0–30 min’ group, three proteins were structurally confirmed to possess GPCR characteristics according to at least two of the software tools, while in the ‘30 min–5 h’ group, two proteins were similarly confirmed (Table 5, Supplementary Files S2–S4). All three of the identified GPCR genes were up-regulated in the ‘0–30 min’ group, whereas evm.TU.ctg441.12 was down-regulated and evm.TU.ctg179.30 was up-regulated in the ‘30 min–5 h’ group.

### 3.6. Time-Series Analysis of IDEGs

**Figure 10:**
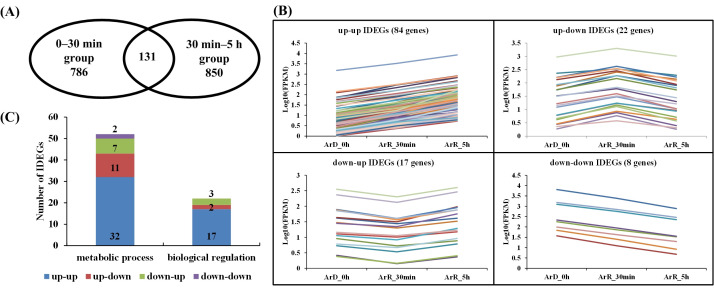
Time-series analysis of the common IDEGs in the 5 h after DET. (**A**) Number of common IDEGs in ‘0–30 min’ and ‘30 min–5 h’ groups. (**B**) Expression profiles of common IDEGs in up–up, up–down, down–up, and down–down groups. The different colors of lines respresent different genes. (**C**) Distribution of IDEGs in metabolic process and biological regulation process in up–up, up–down, down–up, and down–down groups.

The analysis of the ‘0–30 min’ and ‘30 min–5 h’ groups reflected the differential expression of genes in different stages of EDT. In order to further investigate the continuous expression of genes during the 5 h after DET, a time-series analysis of the common IDEGs in the two stages was performed. The ‘0–30 min’ and ‘30 min–5 h’ groups contained 786 and 850 IDEGs, respectively. The common IDEGs in the two groups were extracted and 131 genes were finally found (Figure 10A). The small numbers of common IDEGs further proved the significant differences in metabolic and regulation processes between these two stages. According to differential expression patterns, these genes were divided into four groups: genes up-regulated in both the ‘0–30 min’ and ‘30 min–5 h’ groups (up–up IDEGs); genes up-regulated in the ‘0–30 min’ group but down-regulated in the ‘30 min–5 h’ group (up–down IDEGs); genes down-regulated in the ‘0–30 min’ group but up-regulated in the ‘30 min–5 h’ group (up–down IDEGs); and genes down-regulated in both the ‘0–30 min’ and ‘30 min–5 h’ groups (down–down IDEGs). The expression profiles of the IDEGs in the four groups are displayed in Figure 10A,B. GO enrichment analysis was performed on the genes in these four groups, respectively. Most genes were enriched in “metabolic process” (GO:0008152) and “biological regulation” (GO:0065007). In terms of the genes involved in “biological regulation”, there were a total of 22 genes, with the majority distributed in the up–up group (17 genes). The up–down and down–up groups each only contained two and three genes, respectively, while the down–down group did not contain any regulatory genes. This suggests that over time, the majority of regulatory genes with continuously changing expression are up-regulated, indicating a gradual activation of cellular regulation during the EDT process. Among the 17 regulation related genes in the up–up group, 6 (evm.TU.ctg33.11, evm.TU.ctg470.4, evm.TU.ctg275.1, evm.TU.ctg470.6, evm.TU.ctg756.7, evm.TU.ctg658.6) are involved in the signal transduction process.

## 4. Discussion

### 4.1. Regulation of EDT Process

Embryonic diapause is a fascinating and widely observed biological phenomenon observed in various animal species, particularly invertebrates and some mammals [31]. It serves as a remarkable survival strategy, enabling developing organisms to endure adverse conditions by entering a state of suspended animation [32]. The widespread distribution of embryonic diapause suggests its ancient evolutionary origin and potential shared molecular basis. During diapause, the embryo essentially enters a period of dormancy or quiescence, characterized by significantly reduced metabolic activity and halted development [33].

The initiation and termination of diapause are closely related to environmental factors, but their molecular mechanisms remain unclear. In this study, ATAC-seq and RNA-seq sequencing were conducted on *Artemia* cyst samples at 30 min after EDT, and the results were compared with the sequencing results from cyst samples at the diapause stage and 5 h after EDT to explore the regulatory mechanisms of EDT. During comparative analysis of high-throughput sequencing data at different time points, we observed significant differences in gene expression levels during the first 30 min and at 30 min to 5 h after EDT. By integrating ATAC-seq and RNA-seq sequencing results, 786 and 850 IDEGs were identified in the ‘0–30 min’ and ‘30 min–5 h’ groups, respectively. However, there were only 131 common IDEGs between these two groups, with only 92 IDEGs showing consistent up-/down-regulation patterns. This suggests significant alterations in metabolism and regulation processes within *Artemia* cyst cells during the first 30 min and the subsequent 30 min to 5 h after EDT, with notable differences in the mechanisms underlying these changes. Among the DEGs exhibiting consistent patterns in both stages, there were more genes related to metabolic processes than regulation processes (34 vs. 17), indicating that changes in regulation processes are more pronounced than metabolic processes during the first 30 min and the subsequent 30 min to 5 h after EDT. Among the regulation-related genes, there were 60 and 66 signaling genes in the two stages, respectively, with only 8 of them being the same. Furthermore, when comparing results for the GPCR signaling pathway, which played a dominant role in both groups, it was found that the genes involved in this pathway in the two groups were entirely different. This suggests that the mechanisms of signal transduction processes may be completely different in these two periods. A more detailed stage division should be carried out in future research to deeply analyze the dynamic regulation process of EDT.

In both the ‘0–30 min’ and ‘30 min–5 h’ groups, signaling regulation was mainly mediated through the GPCR signaling pathway and cell surface receptor signaling pathway. In the ‘0–30 min’ group, hormone-mediated signaling pathways also played important roles, which was distinct from the ‘30 min–5 h’ group. In terms of other signaling pathways, both groups involved enzyme-linked receptor protein signaling pathways and Wnt and Notch signaling pathways. However, the specific genes involved in these pathways differed between the two groups. Additionally, the ‘0–30 min’ group included the tachykinin receptor signaling pathway and the neurotrophin signaling pathway, while the ‘30 min–5 h’ group included the neuropeptide signaling pathway. These results indicated that hormones play a crucial role in the first 30 min of EDT. In the 5 h following EDT, classical signaling pathways such as GPCR, Wnt, and Notch are then involved. However, the genes involved in these pathways vary between different time periods. The relationship between the Wnt signaling pathway and EDT is consistent with the findings of Jia et al. [10]. Furthermore, Ouellet et al.’s study [34] also suggests that the Notch signaling pathway is associated with the maintenance and termination of diapause in *C. elegans* embryos. Additionally, we propose that the ‘0–30 min’ and ‘30 min–5 h’ stages contain their own unique signaling regulation pathways.

Based on the time-series analysis of the common IDEGs between the ‘0–30 min’ and ‘30 min–5 h’ groups, most metabolic and regulated related genes were found in the up–up group, indicating that metabolic and regulation processes were activated during the 5 h after EDT. As there were more IDGEs in the ‘30 min–5 h’ group compared to the ‘0–30 min’ group, we surmise that this activation is a gradual process and peaks at 5 h after EDT, which is consistent with the research of Chen et al. [15] and Yu et al. [16]. In the six signal-transduction-related genes, evm.TU.ctg33.11 is a GTPase-activating protein (GAP) for RhoA/Rho1, which plays an essential role in the regulation of the RhoA/Rho1-Drok-MRLC signaling pathway [35]. evm.TU.ctg470.4 is an epidermal growth factor receptor gene that is involved in developmental decisions by transducing signals through the ras-raf-MAPK pathway. evm.TU.ctg275.1 participates in the proton transfer required for signaling transduction through proton-selective channels [36]. evm.TU.ctg756.7 encodes a regulator of G-protein signaling, Loco, which is required for dorsal/ventral axis formation of the egg and embryo [37].

### 4.2. GPCRs Participating in Artemia EDT Process

The GPCR, also known as a seven-transmembrane receptor or heptahelical receptor, is a type of cell membrane receptor protein that spans the cell membrane seven times, forming a distinctive helical structure. GPCRs constitute a diverse and extensive family of proteins present in the cell membranes of various organisms, including human [38], mouse [39], and *Takifugu rubripes* [40]. These receptors play a fundamental role in cellular signal transduction, serving as mediators for transmitting signals from the extracellular environment into the interior of the cell. They are crucial in facilitating the cellular response to a wide range of signals, including neurotransmitters, hormones, ions, and even light [41,42]. In insects, GPCRs have been found to interact with diapause hormones as ligands, suggesting their potential involvement in the signaling transduction of the diapause process [43,44].

In this study, we identified 5 and 10 genes with GPCR activity (GO:0004930) in the ‘0–30 min’ and ‘30 min–5 h’ groups, respectively. To further confirm whether these genes encode GPCR proteins, the secondary and tertiary structures of the proteins encoded by them were analyzed using three tools: TMHMM, PredictProtein, and SWISS-MODEL. Based on the distinctive 7-TMH structural characteristic of GPCRs, it was determined that three genes (evm.TU.ctg485.29, evm.TU.ctg71.25, evm.TU.ctg321.8) in the ‘0–30 min’ group and two genes (evm.TU.ctg441.12, evm.TU.ctg179.30) in the ‘30 min–5 h’ group encode proteins with structural characteristics consistent with GPCRs. evm.TU.ctg485.29, evm.TU.ctg71.25, and evm.TU.ctg321.8 were all found to be up-regulated in the ‘0–30 min’ group, suggesting that they are involved in processes occurring during the initial 30 min after EDT. evm.TU.ctg441.12 was found to be down-regulated exclusively in the ‘30 min–5 h’ group, indicating its potential significance as a GPCR during both the diapause stage and the 30 min period after EDT. Additionally, evm.TU.ctg179.30 exhibited up-regulation in the ‘30 min–5 h’ group, suggesting its primary function occurs in the 5 h after EDT.

In the genome annotation of *A. franciscana*, evm.TU.ctg485.29 was identified as an Adhesion GPCR E5, a member of the LN-TM7 subfamily of GPCRs. This receptor is potentially involved in both adhesion and signaling processes shortly after leukocyte activation, playing a crucial role in leukocyte migration. evm.TU.ctg321.8 was annotated as Frizzled-10, a receptor for Wnt proteins. It has been shown to function in the canonical Wnt/beta-catenin signaling pathway and is activated by WNT7A to induce the expression of beta-catenin target genes, as observed in *Gallus gallus* [45]. It belongs to the Fz/Smo family of GPCRs. evm.TU.ctg441.12 is a RYamide receptor belonging to the GPCR1 family, serving as a receptor for the neuropeptides RYamide-1 and RYamide-2 in *Drosophila melanogaster* [46,47]. evm.TU.ctg179.30 falls within the Opsin family of GPCRs, which are integral components of visual pigments. Visual pigments are molecules responsible for light absorption and mediating vision. They consist of an apoprotein, opsin, covalently linked to 11-cis-retinal [48].

## 5. Conclusions

Embryonic diapause is a widely observed evolutionary adaptation phenomenon in various organisms, and yet the mechanisms governing its initiation and termination remain unclear. In this study, we found significant changes in the metabolism and regulation of cyst cells during the initial 30 min and the subsequent 30 min to 5 h after diapause termination. The synthesis of certain diapause-related metabolites and hormone-mediated regulation appeared to be more active within the first 30 min. GPCR and cell surface receptor signaling pathways play a dominant role in signal regulation during EDT, but the specific genes and signaling mechanisms engaged in the first 30 min and the subsequent stages are entirely distinct. This is specifically manifested in different GPCRs active in different stages. In this work, three and two different GPCR genes were identified in the ‘0–30 min’ and ‘30 min–5 h’ groups, respectively. The results of this work provide valuable insights for further research into the regulatory mechanisms underlying diapause and the EDT process in *Artemia* cysts. The concrete function of the signal transduction pathways and GPCRs involved in EDT still needs to be verified in further experiments. In addition, the specific correspondence between different GPCRs and signal transduction pathways is also an interesting topic for future research, which may help researchers to reconstruct the complete signaling transfer chain within cells for the EDT process.

## Figures and Tables

**Table 1:** Results of data trimming and quality control.

Sample	Raw_Reads	Clean_Reads	Clean_Ratio	Q20	Q30	Trimmed_with_Adapter
ArR_30min_1	52,357,910	48,803,784	76.96%	96.38%	90.97%	45,083,296
ArR_30min_2	57,169,025	53,476,918	75.86%	96.17%	90.35%	53,291,712
ArR_30min_3	84,056,461	78,227,165	75.02%	96.45%	91.05%	79,721,582

**Table 2:** Results of mapping to reference genome and peak calling.

Sample	Mapped ^1^	Unique_Mapped ^2^	Unique_Mapped_Dedup ^3^	Peak	Summits
ArR_30min_1	45,415,648 (93.06%)	31,888,289 (70.21%)	26,502,687 (54.30%)	15,449	16,887
ArR_30min_2	49,757,224 (93.04%)	34,420,807 (69.18%)	28,434,872 (53.17%)	11,741	12,837
ArR_30min_3	73,026,077 (93.35%)	48,899,473 (66.96%)	41,214,444 (52.69%)	62,877	74,549

^1^ Number of mapped reads (the proportion in parentheses is the percentage of mapped reads relative to clean reads). ^2^ The number of reads with unique alignment positions on the reference sequence (the proportion in parentheses is the percentage of unique_mapped reads relative to clean reads). ^3^ The number of reads after removing the duplicate reads mapping to a unique reference sequence (the proportion in parentheses is the percentage of unique_mapped_dedup reads relative to clean reads).

**Table 3:** Results of RNA-seq.

Sample	Clean Reads	Q20	Q30	GC	Mapped ^1^	Unique_Mapped ^2^
ArR_30min_1	54,938,820	97.9	94.07	39.67	49,010,515 (89.21%)	48,517,386 (88.31%)
ArR_30min_2	41,809,618	97.88	94.04	39.74	37,406,369 (89.47%)	37,033,552 (88.58%)
ArR_30min_3	46,087,654	97.54	93.19	39.87	40,977,822 (88.91%)	40,566,401 (88.02%)

^1^ Number of mapped reads (the proportion in parentheses is the percentage of mapped reads relative to clean reads). ^2^ The number of reads with unique alignment positions on the reference sequence (the proportion in parentheses is the percentage of unique_mapped reads relative to clean reads).

**Table 4:** Comparison of DEGs from ATAC-seq and RNA-seq.

Group	Subject	Up-Regulated	Down-Regulated	Total
‘0–30 min’	ATAC-seq	1880	2831	4261
	RNA-seq	2414	1889	4303
	Common	834	363	1197
	IDEGs	579	207	786
‘30 min–5 h’	ATAC-seq	2784	1528	4312
	RNA-seq	2999	2816	5815
	Common	824	795	1619
	IDEGs	561	289	850

**Table 5:** Prediction of TMHs for the candidate GPCR genes.

Group	Candidate GPCR Genes	TMHMM	PredictProtein	SWISS-MODEL
‘0–30 min’	evm.TU.ctg485.29	7	7	7
	evm.TU.ctg71.25	7	7	7
	evm.TU.ctg321.8	7	7	7
	evm.TU.ctg288.3	5	5	5
	evm.TU.ctg308.1	0	0	6
‘30 min–5 h’	evm.TU.ctg441.12	7	7	7
	evm.TU.ctg179.30	6	7	7
	evm.TU.ctg445.18_evm.TU.ctg445.12	4	5	5
	evm.TU.ctg195.9	4	4	5
	evm.TU.ctg42.32	4	4	4
	evm.TU.ctg41.1	4	3	0
	evm.TU.ctg1716.2	2	5	2
	evm.TU.ctg25.48	0	0	0
	evm.TU.ctg115.32	0	0	0
	evm.TU.ctg179.3	0	0	0

## Data Availability

The ATAC-seq and RNA-seq data have been deposited in the GEO database with the accession numbers GSE254934 and GSE254935.

## Supplementary Materials

The following supporting information can be downloaded at https://www.mdpi.com/article/10.3390/cimb46040229/s1, Supplementary File S1, The GO annotations of IDEGs in the ‘0–30 min’ and ‘30 min–5 h’ groups. Supplementary File S2, The structure of candidate GPCRs predicted by TMHMM. Supplementary File S3, The structure of candidate GPCRs predicted by PredictProtein. Supplementary File S4, The structure of candidate GPCRs predicted by SWISS-MODEL.

## Abbreviations

EDT	Embryonic diapause termination
DEGs	Differentially expressed genes
TSS	Downstream gene start site
GPCR	G-protein-coupled receptor
TMH	Transmembrane helix
