# Supplementary figures and images for: Signaling Transduction Pathways and G-Protein-Coupled Receptors in Different Stages of the Embryonic Diapause Termination Process in Artemia
Source: Curr Issues Mol Biol. 2024 Apr 20;46(4):3676–93. doi: 10.3390/cimb46040229 (PMC11049050; doi:10.3390/cimb46040229)

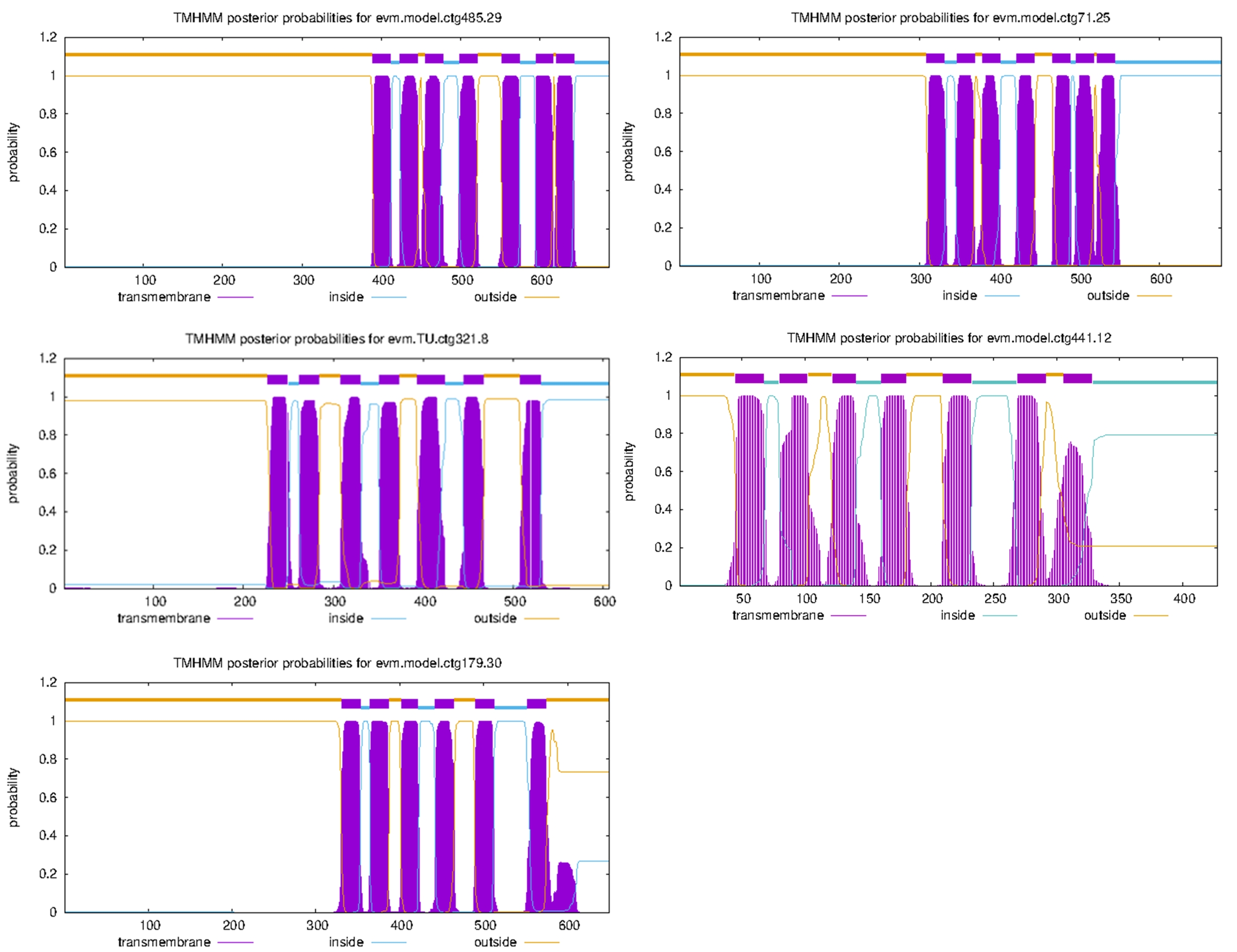

Supplement: Supplementary file 1 [file cimb-46-00229-s001.zip › Supplementary file S2.jpg]

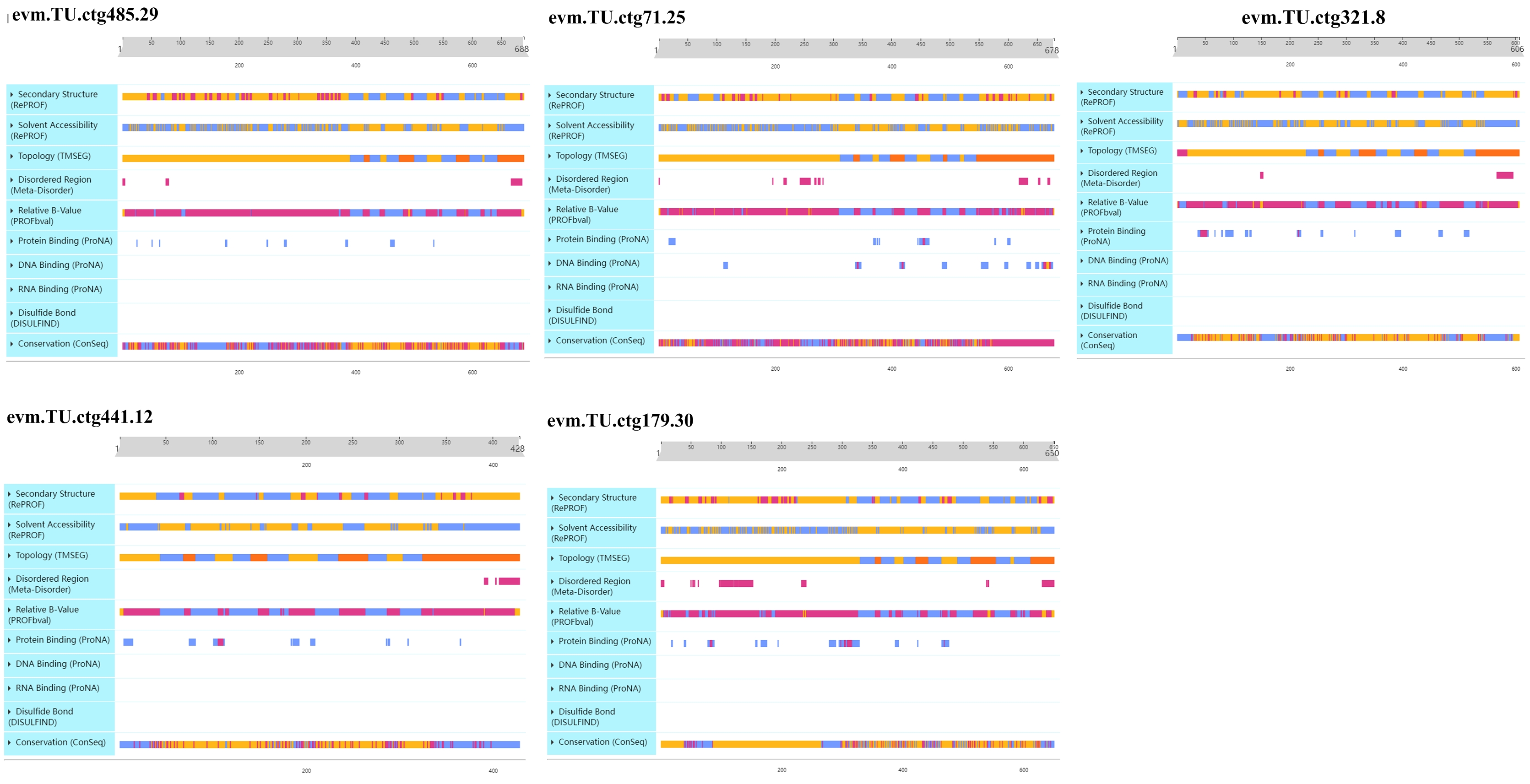

Supplement: Supplementary file 1 [file cimb-46-00229-s001.zip › Supplementary file S3.jpg]

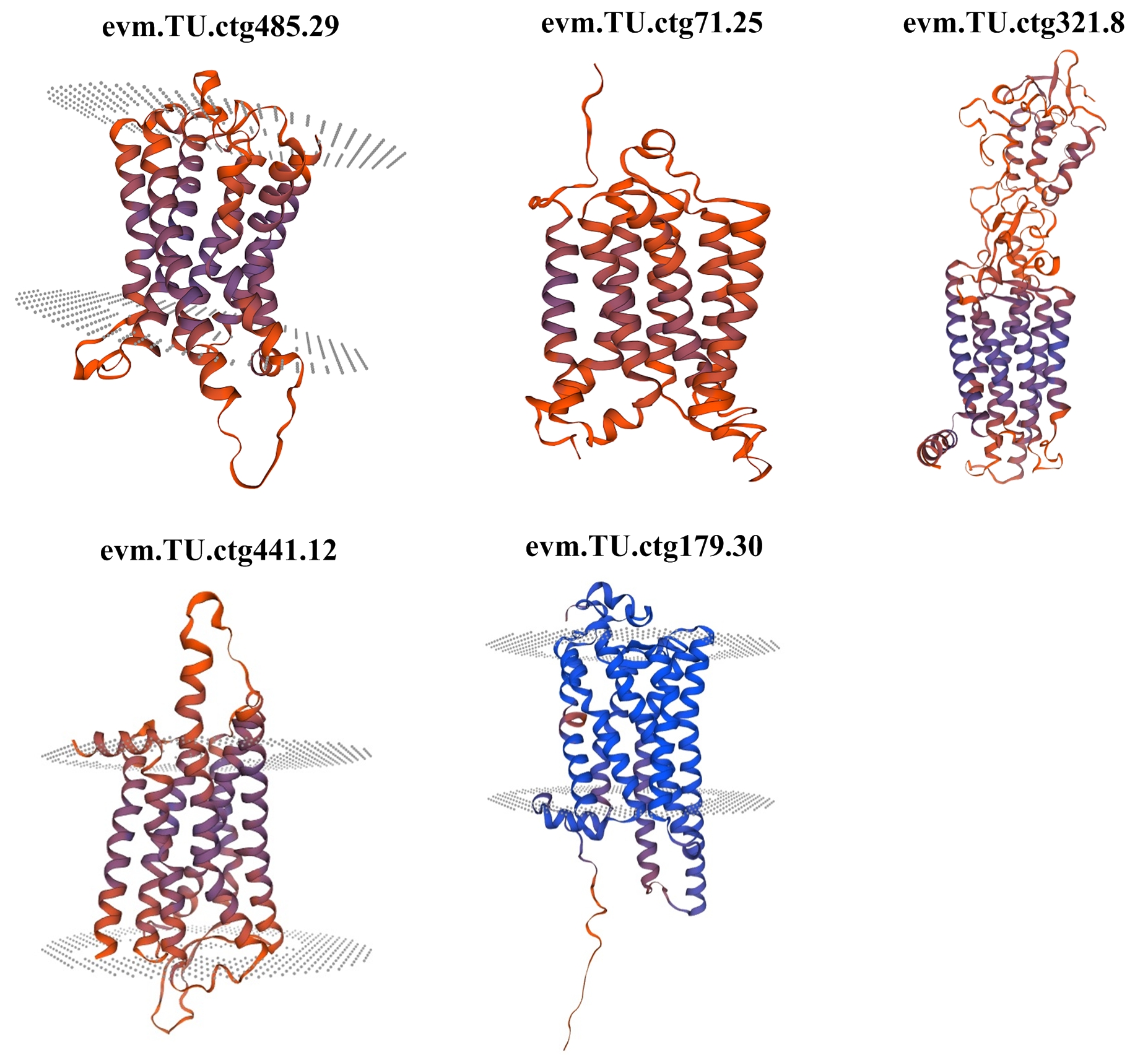

Supplement: Supplementary file 1 [file cimb-46-00229-s001.zip › Supplementary file S4.jpg]
